# Polymeric Nanogels for Skin Applications

**DOI:** 10.3390/gels12050354

**Published:** 2026-04-23

**Authors:** Sara Silva, Manuela Machado, Eduardo M. Costa

**Affiliations:** Universidade Católica Portuguesa, CBQF—Centro de Biotecnologia e Química Fina—Laboratório Associado, Escola Superior de Biotecnologia, Rua Diogo Botelho 1327, 4169-005 Porto, Portugal; snsilva@ucp.pt (S.S.); mmachado@ucp.pt (M.M.)

**Keywords:** polymeric nanogels, skin inflammatory diseases, psoriasis, atopic dermatitis, stimuli-responsive, nanozymes, immunomodulation, transdermal drug delivery

## Abstract

Chronic skin inflammatory diseases including psoriasis and atopic dermatitis affect millions worldwide, imposing substantial physical, psychological, and economic burdens. Despite advances in topical therapies, conventional formulations suffer from poor skin penetration, rapid clearance, local and systemic side effects, and suboptimal patient adherence. Polymeric nanogels, internally crosslinked three-dimensional polymer networks with dimensions of 10–200 nm, emerged as promising platforms to overcome these limitations. Their unique properties including high water content, tunable porosity, biocompatibility, deformability, and stimulus-responsive behavior enhance skin penetration allowing for targeted therapeutic action. This review examines nanogel synthesis methods optimized for targeting skin inflammatory diseases, including biopolymer-based approaches utilizing chitosan and hyaluronic acid, offering insights into how different methods and advanced architecture provide multifunctional capacities and bioactivities. Translation challenges including manufacturing scalability, long-term safety assessment, and regulatory compliance are critically discussed alongside emerging opportunities in personalized medicine and smart microneedle integrated systems for adaptive therapy.

## 1. Introduction

Chronic skin inflammatory diseases, including psoriasis and atopic dermatitis, affect 20 to 25% of world population, impacting up to 20% of children, and represent a prevalent and burdensome dermatological conditions [1]. These diseases impose substantial physical costs through painful lesions and pruritus, psychological impacts including depression and social isolation, and economic burdens through direct medical expenses and indirect productivity losses [2]. Despite their vast immunological profiles, these conditions share common pathophysiological features creating therapeutic challenges and opportunities for innovative delivery approaches [3,4]. Complex immune dysregulation involves multiple cell types including T lymphocytes, dendritic cells, macrophages, mast cells, and innate lymphoid cells orchestrating inflammatory cascades [5]. Additionally, impaired skin barrier function represents another shared feature with profound therapeutic implications, manifesting through disrupted stratum corneum organization with altered lipid lamellae structure, reduced ceramide content particularly in atopic dermatitis where levels may decrease 30–50% compared to healthy skin, increased corneocyte spacing, compromised tight junctions, and overall degradation of barrier brick-and-mortar architecture [6,7].

### 1.1. Limitations of Conventional Topical Therapies

Conventional topical therapies despite decades of clinical use suffer from fundamental limitations compromising therapeutic outcomes [8]. Topical corticosteroids representing first line therapy achieve anti-inflammatory effects through NF-κB suppression, phospholipase A2 inhibition reducing arachidonic acid release, lysosomal membrane stabilization, and inflammatory cell migration inhibition [9,10]. However, clinical utility is constrained by poor skin penetration particularly of potent corticosteroids, with typically <1–5% of applied dose reaching viable epidermis and dermis where therapeutic targets reside, necessitating high concentrations and frequent application. Rapid clearance through cutaneous enzyme metabolism, diffusion back into circulation, and mechanical removal through desquamation results in short residence times requiring twice or more daily applications [11,12].

This combination creates narrow therapeutic windows where underdosing fails to control inflammation while chronic use causes well-documented adverse effects. Local side effects include skin atrophy manifesting as epidermal and dermal thinning with subcutaneous fat loss, telangiectasia from weakened vessel walls, striae from dermal collagen destruction, increased infection susceptibility, delayed wound healing, and rebound flares upon discontinuation [13,14,15].

### 1.2. The Rationale for Hydrogel-Based Delivery Systems

Hydrogels—three-dimensional crosslinked polymer networks capable of absorbing large quantities of water while maintaining structural integrity—have emerged as exceptionally promising platforms for topical drug delivery, offering unique properties directly addressing conventional formulation limitations [16]. The fundamental hydrophilic nature from abundant polar functional groups including hydroxyl, carboxyl, amide, and amine groups enables water uptake ranging from several hundred to over 1000% of dry polymer weight depending on composition, crosslinking density, and ionic strength [17]. This high water content creates aqueous microenvironments closely mimicking hydrated healthy skin and wound beds, proving highly compatible with sensitive or compromised skin that may not tolerate oil-based ointments or alcohol-containing solutions, providing cooling and soothing sensations alleviating burning, stinging, or pruritus common in inflammatory conditions [18,19]. Furthermore, the porous structure with mesh sizes controllable through polymer molecular weight, crosslinking density, and swelling conditions enables efficient drug loading through simple diffusion or incorporation during synthesis, accommodates molecules across wide size ranges from small drugs through macromolecular therapeutics including peptides, proteins, antibodies, and nucleic acids, and facilitates controlled release through diffusion with tunable kinetics [20,21]. Additionally, biocompatibility particularly for natural polymer-based hydrogels derived from hyaluronic acid, chitosan, alginate, and cellulose derivatives or proteins including gelatin and collagen exhibits minimal cytotoxicity, low immunogenicity, and often intrinsic bioactive properties supporting wound healing and skin regeneration [22].

Another factor to consider is the hydrogels’ mechanical properties. Being highly tunable enables formulations ranging from soft fluid gels for comfortable spreading to firm elastic gels maintaining position in challenging anatomical sites, with viscoelastic behavior providing both solid-like resistance maintaining structural integrity and liquid-like flow during application, spreading thin layers easily [23,24]. The potential for stimulus-responsive behavior represents perhaps the most therapeutically valuable attribute for inflammatory diseases where pathological microenvironments create exploitable differences from healthy tissue [25].

### 1.3. From Macroscopic Hydrogels to Nanohydrogels

While macroscopic hydrogels offer advantages including enhanced hydration, controlled release, and improved patient acceptance, their ability to actively penetrate through the stratum corneum into the viable epidermis and dermis where inflammatory processes occur remains limited by macroscopic dimensions [26]. The stratum corneum consists of 10 to 20 layers of dead keratinized corneocytes measuring 30–40 μm in diameter and 0.5–1.0 μm in thickness. It creates a tortuous pathway which permits passive diffusion only of molecules meeting strict criteria, including molecular weight generally <500 Da, octanol-water partition coefficients approximately 1–3 balancing lipophilicity and hydrophilicity, and limited hydrogen bonding capability [27,28]. Within this framework, the transition from macroscopic to nanohydrogels (crosslinked three-dimensional polymer networks with characteristic dimensions typically 10–200 nm) fundamentally transforms therapeutic potential by enabling the carrier itself to penetrate through skin barriers and accumulate in target tissues (Table 1) [29,30].

### 1.4. Advanced Nanohydrogel Architectures for Inflammatory Disease Therapy

**Figure 1:**
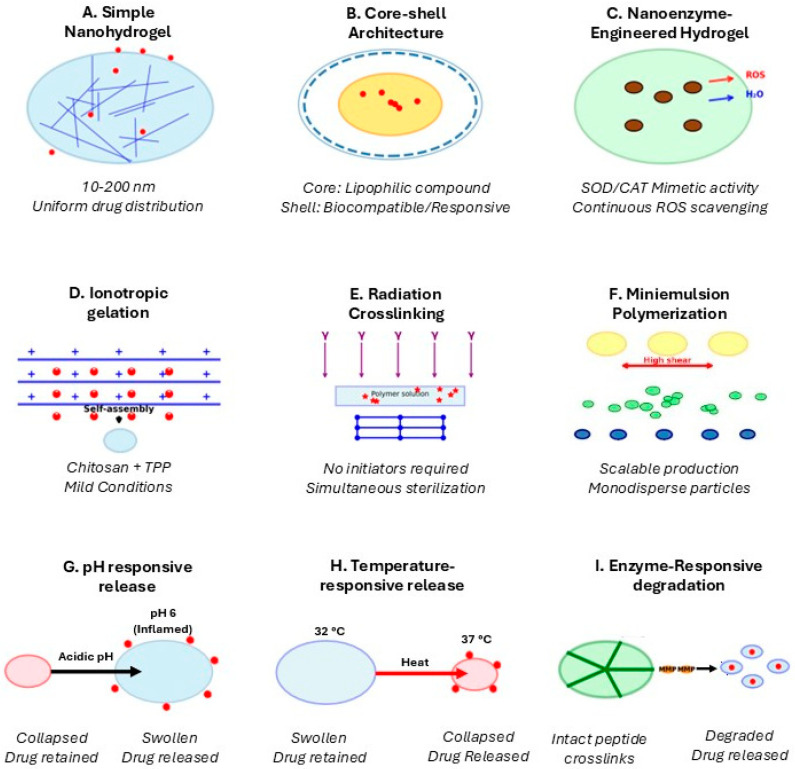
Polymeric nanohydrogel architectures and synthesis strategies for inflammatory skin applications, including core-shell systems (80–200 nm), IPN networks, and nanozyme-integrated designs for ROS-responsive delivery in inflammatory skin conditions.

Recent advances in nanohydrogel synthesis have produced increasingly sophisticated systems specifically tailored for inflammatory skin disease applications (Figure 1).

Core–shell nanohydrogels featuring distinct compositional domains with hydrophobic or drug-loaded cores surrounded by hydrophilic biocompatible shells provide multiple advantages [35]. The core can be engineered for efficient encapsulation of lipophilic anti-inflammatory drugs including corticosteroids, retinoids, or natural compounds like curcumin achieving drug loading capacities for hydrophobic drugs such as curcumin and betamethasone which frequently reach 30–40% (*w*/*w*) via nanoprecipitation into hydrophobic PLGA or PCL cores, compared with 10–15% (*w*/*w*) typically achieved in homogeneous chitosan or poly(NIPAM) nanohydrogels loaded by equilibrium swelling diffusion [36]. The shell serves critical functions including colloidal stability through steric or electrostatic repulsion, drug protection from premature release or degradation, controlled release kinetics through tunable shell thickness and permeability, and biocompatibility through incorporation of PEG or zwitterionic polymers reducing protein adsorption [37]. Stimuli-responsive shells prove particularly valuable where properties change in response to pathological microenvironment cues. pH-responsive shells incorporating ionizable groups remain intact at normal skin pH level (~5.5 surface, 7.4 in viable tissue) but swell dramatically when encountering altered pH in inflammatory conditions [38]. Temperature-responsive shells based on poly(N-isopropylacrylamide) remain swollen at normal skin temperature but collapse upon encountering elevated temperature of inflamed tissue [39]. Enzyme-responsive shells incorporating peptide crosslinks or polymers degradable by matrix metalloproteinases dramatically elevated in chronic inflammation respond to high enzyme concentrations by shell degradation triggering burst release specifically in high-protease environments [40].

Other developments are nanozyme-engineered nanohydrogels, which are a particularly promising avenue for inflammatory diseases where oxidative stress plays central pathogenic roles. Nanozymes, nanoscale materials mimicking natural enzyme catalytic activities—including superoxide dismutase, catalase decomposing hydrogen, peroxidase reducing hydrogen peroxide, or oxidase catalyzing substrate oxidation—can be integrated into nanohydrogel networks, and downregulate MAPK and NF-κB signaling pathways, reducing pro-inflammatory cytokine production [41,42].

## 2. Synthesis Methods for Inflammatory Disease Applications

The synthesis method profoundly influences nanohydrogel properties and biological performance through effects on architectural features, network microstructure, surface characteristics, and responsive behaviors. Selection of appropriate synthesis approaches depends on intended application requirements including drug loading capacity, release kinetics, penetration routes, and responsiveness to inflammatory microenvironments [43].

### 2.1. Biopolymer-Based Synthesis

#### 2.1.1. Chitosan-Based Nanohydrogels

Chitosan-based nanohydrogels, derived from chitin deacetylation, provide therapeutic advantages beyond drug delivery due to their intrinsic antimicrobial, mucoadhesive, and wound-healing properties [44]. Their polycationic structure enables electrostatic interactions with negatively charged bacterial membranes, causing membrane disruption and antimicrobial activity that is particularly valuable in inflammatory skin conditions where barrier dysfunction increases infection risk [45]. Mucoadhesive properties arise from ionic interactions between protonated amine groups and negatively charged biological surfaces, prolonging residence time and enabling sustained drug release [46]. In addition, chitosan promotes tissue regeneration by stimulating immune cell activation, fibroblast proliferation, collagen synthesis, angiogenesis via VEGF upregulation, and keratinocyte migration during re-epithelialization [47]. Chitosan nanohydrogels are commonly synthesized through:

1. Ionotropic gelation with anionic crosslinkers such as tripolyphosphate, forming nanoparticles through electrostatic interactions [48];
2. Polyelectrolyte complexation with anionic polymers (e.g., alginate, gellan gum, hyaluronic acid) generating stable interpolymer networks [49];
3. Chemical crosslinking using agents such as genipin to enhance structural stability under physiological conditions [50].

Polyelectrolyte complexation is not limited to biopolymer pairs. Synthetic polyelectrolyte systems offer complementary advantages, including more precise control over charge density, molecular weight, and functionalization [51]. Representative synthetic PEC nanohydrogel systems include poly(acrylic acid) (PAA)/poly(allylamine hydrochloride) (PAH) complexes, which form stable nanoparticles through strong electrostatic interactions across a wide pH range [52], and poly(styrene sulfonate) (PSS)/poly(diallyldimethylammonium chloride) (PDADMAC) pairs, which produce nanohydrogels with tunable surface charge for skin deposition [53]. Compared to biopolymer-based PEC systems, synthetic PEC nanohydrogels permit more reproducible batch-to-batch formation through precise monomer ratio control, but generally lack the intrinsic bioactivity (antimicrobial properties, wound-healing promotion, CD44-receptor targeting) characteristic of chitosan or hyaluronic acid counterparts [54]. The choice between synthetic and biopolymer-derived PEC systems should therefore be guided by whether intrinsic bioactivity or manufacturing reproducibility is the primary design criterion.

#### 2.1.2. Hyaluronic Acid-Based Nanohydrogels

Hyaluronic acid (HA) nanohydrogels offer advantages due to HA’s natural presence in the dermal extracellular matrix, where it contributes to tissue hydration, viscoelasticity, and cell signaling [55]. HA interacts with CD44 receptors expressed on keratinocytes, macrophages, and activated immune cells, enabling receptor-mediated targeting in inflammatory conditions [56]. HA also exhibits intrinsic anti-inflammatory activity through free radical scavenging, sequestration of pro-inflammatory mediators, and modulation of immune cell behavior through CD44 signaling pathways [57]. Its exceptional hygroscopicity—binding water up to 1000 times its own weight—creates highly hydrated nanohydrogels that are particularly beneficial for xerotic inflammatory skin disorders [58]. HA nanohydrogels can be produced via chemical crosslinking (e.g., carbodiimide-mediated coupling or divinyl sulfone reactions) or self-assembly strategies such as cholesterol-modified HA forming nanogels through hydrophobic interactions [59].

### 2.2. Radiation-Induced Crosslinking

Radiation-induced crosslinking uses ionizing radiation (e.g., gamma rays or electron beams) to generate nanogels without chemical initiators or crosslinkers. This additive-free approach produces highly pure materials while simultaneously achieving terminal sterilization due to radiation’s bactericidal and virucidal effects [60]. Crosslinking occurs through direct polymer radical formation or indirect reactions following water radiolysis, producing reactive species that induce intramolecular recombination [61]. In dilute polymer solutions (0.1–5% *w*/*v*), intramolecular crosslinking predominates, generating discrete nanogels rather than bulk networks. Nanogel properties depend on parameters such as radiation dose, dose rate, temperature, and oxygen removal to prevent oxidative degradation. Lower doses (5–10 kGy) favor loosely crosslinked, high-swelling nanogels, while higher doses (25–50 kGy) yield denser networks and additionally fulfill sterilization requirements [62]. This method provides exceptional purity, guaranteed sterility with sterility assurance levels exceeding 10^−6^, and excellent biocompatibility with cell viabilities often exceeding 95% [63].

### 2.3. Emulsion Polymerization Approaches

Emulsion polymerization enables scalable production of monodisperse nanohydrogels with high batch reproducibility [64]. Miniemulsion polymerization is particularly effective, using high-shear homogenization to generate monomer droplets (50–500 nm) stabilized by surfactants and co-stabilizers [65]. Each droplet functions as an individual nanoreactor where polymerization occurs independently, preserving droplet size distribution and allowing precise control of particle size [66]. This method is especially suitable for encapsulating hydrophobic anti-inflammatory drugs such as corticosteroids, retinoids, or curcumin, achieving loading capacities above 30–40% by weight [67]. However, surfactant removal through purification processes such as dialysis or diafiltration is necessary to prevent skin irritation, requiring careful analytical verification of residual levels [68].

### 2.4. Photoinitiated Polymerization

Photoinitiated polymerization provides spatiotemporal control over nanogel synthesis through light-activated initiation at ambient temperatures, enabling compatibility with heat-sensitive molecules and integration with advanced fabrication techniques such as 3D printing [69]. Type I photoinitiators (e.g., benzoin derivatives) generate radicals through direct cleavage, producing rapid polymerization and highly crosslinked networks, whereas Type II systems (e.g., benzophenone) rely on bimolecular hydrogen abstraction mechanisms that enable more controlled polymerization [70]. Emerging photoinitiator systems, including conjugated polymer nanoparticles, offer high extinction coefficients, oxygen tolerance, and multifunctionality, enabling both crosslinking and antibacterial activity [71]. Visible-light photoinitiators (400–700 nm) are particularly advantageous due to deeper tissue penetration, reduced cytotoxicity compared with UV radiation, and compatibility with low-cost LED light sources [72]. Photoinitiators cytotoxicity is a key safety consideration for topical applications. I2959 (2-hydroxy-4′-(2-hydroxyethoxy)-2-methylpropiophenone), one of the most widely used UV photoinitiators in hydrogel synthesis, exhibited no cytotoxicity towards dermal fibroblasts at 0.5% (*w*/*v*) with 24 h of irradiation. On the other hand, lithium phenyl-2,4,6-trimethylbenzoylphosphinate (LAP), a visible-light photoinitiatior, significantly reduced fibroblast viability at 1% (*v*/*v*) with active concentrations needing to be reduced to either 0.1 and 0.025% (*v*/*v*) to meet biocompatibility requirements [73]. The main characteristics, advantages, and limitations of the nanohydrogel systems and synthesis strategies described in this section are summarized in Table 2.

## 3. Mechanisms of Action and Clinical Applications

### 3.1. Pathophysiological Targeting in Inflammatory Skin Diseases

**Figure 2:**
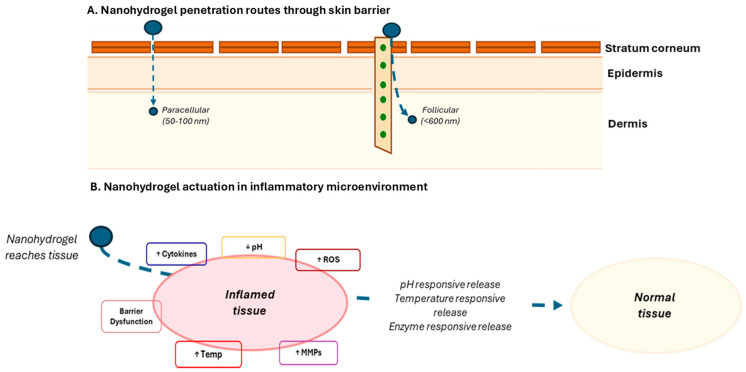
Examples of nanohydrogel skin penetration routes and targeting of inflammatory microenvironment in skin diseases.

Nanohydrogels address inflammatory skin disease pathophysiology through multiple complementary mechanisms operating at molecular, cellular, and tissue levels. The inflammatory microenvironment exhibits characteristic alterations exploitable for selective targeting and triggered release (Figure 2) [74]. Elevated reactive oxygen species in inflamed skin reaching concentrations 10–100-fold higher than normal tissue can be exploited through incorporation of oxidation-sensitive linkages or radical-scavenging moieties. Nanozyme-engineered hydrogels mimicking SOD, catalase, and peroxidase activities provide continuous ROS scavenging, reducing oxidative damage to cellular membranes, proteins, and DNA while simultaneously modulating redox-sensitive signaling pathways including NF-κB and AP-1 [75].

Three major chemical linkage strategies underpin ROS-responsive nanohydrogel design:

1. Thioether and thioketal groups, which are oxidized to sulfoxides and sulfones by H_2_O_2_, triggering a hydrophobic-to-hydrophilic switch which destabilizes the nanohydrogel network and leads to the release of the encapsulated drug [76].
2. Arylboronic acid esters, cleaved by H_2_O_2_ via a peroxyboronic acid mechanism with a half-life of minutes at pathologically relevant H_2_O_2_ concentrations (~100 μM), leading to the release of its cargo [77,78].
3. Diselenide and disulfide bonds, susceptible to cleavage by superoxide radicals or elevated intracellular glutathione concentrations characteristic of oxidatively stressed cells [79,80].

Each linkage type can be matched to the predominant ROS species and the intended release compartment—extracellular for thioketals, intracellular for disulfide-based systems.

Acidic pH shifts in inflamed tissue, with values often being around pH 5.5–6.5 compared to normal surface pH 4.5–5.5 and viable tissue pH 7.0–7.4, which enables pH-responsive systems. Nanohydrogels incorporating ionizable groups like carboxylic acids (pKa~4.5–5.0) or amines (pKa~9–10) undergo dramatic volume transitions at characteristic pH values, remaining collapsed and drug-retentive at normal pH level but swelling extensively when encountering altered inflammatory pH triggering accelerated release. Representative swelling data demonstrate that poly(acrylic acid)-based nanohydrogels exhibit swelling ratios (Q) of 5–15 at pH 7.4 versus less than 2 at pH 5.0. Release kinetics fitted to the Korsmeyer-Peppas power law (Mt/M∞ = kt^n^) typically yield exponent n values of 0.45–0.6, indicating anomalous non-Fickian transport dominated by coupled diffusion and polymer chain relaxation [81,82].

Temperature elevations of 1–3 °C characteristic of inflamed skin though appearing modest prove sufficient for triggering phase transitions in temperature-responsive polymers like poly(N-isopropylacrylamide) when LCST is appropriately tuned to ~35–36 °C through copolymer composition [83]. Here, LCST refers to the lower critical solution temperature, the temperature above which thermoresponsive polymers undergo a hydrophilic-to-hydrophobic transition and collapse [84]. Normal skin surface temperature is 32–34 °C, while inflamed psoriatic or eczematous skin displays local elevations of 1–3 °C measured by infrared thermometry in clinical studies, justifying the 35–36 °C target [84]. LCST tuning is achievable by incorporating hydrophilic co-monomers such as acrylamide into poly(NIPAM) to shift the transition temperature upward as required for a given application [85].

Last but not least, dramatically elevated enzymatic activity, particularly matrix metalloproteinases in chronically inflamed skin or non-healing wounds, can exceed normal levels by factors of 100–1000 providing extremely selective triggers. Nanohydrogels incorporating enzyme-cleavable peptide sequences (e.g., Gly-Pro-Leu-Gly-Val-Arg recognized by MMP-2) remain stable in healthy tissue with basal enzyme levels but degrade rapidly in high-protease environments triggering burst drug release [86]. It is important to note, however, that MMP-2 and MMP-9 expression is highly variable across disease stages and patient populations [87]. One particular example is mild psoriasis, where MMP elevation may be only 10–20-fold above baseline, whereas severe chronic plaque psoriasis or acute atopic dermatitis flares may exceed 100–1000-fold [88]. This variability may result in inconsistent trigger efficiency across patients and represents a key limitation of single-stimulus enzyme-responsive designs. Potential mitigation strategies include the use of lower enzyme-threshold cleavage sequences or dual-responsive systems combining enzymatic triggers with pH- or ROS-responsiveness to improve robustness across disease severity spectra.

### 3.2. Immunomodulatory Mechanisms

#### 3.2.1. Macrophage Polarization

Macrophage polarization represents a critical immunomodulatory mechanism for nanohydrogel therapy. Nanozyme hydrogels effectively downregulate MAPK and NF-κB signaling pathways reducing proinflammatory cytokine production including TNF-α, IL-1β, and IL-6 while upregulating anti-inflammatory mediators like IL-10 and TGF-β [89]. This shifts macrophages from pro-inflammatory M1 phenotype, which is characterized by high TNF-α, iNOS expression, and ROS production, towards anti-inflammatory M2 phenotype, characterized by producing IL-10, TGF-β, and arginase, thus supporting tissue repair and regeneration [90,91]. Studies demonstrate that curcumin-loaded nanohydrogels at 10 μg/mL significantly reduced M1 markers (CD86 and iNOS expression by ~40–60%) while increasing M2 markers (CD206 and Arg-1 by ~50–80%) in LPS-stimulated RAW 264.7 macrophages, a result which correlated with atopic dermatitis animal models where reduced clinical scores and improved barrier function were observed [92]. Cerium oxide nanozyme-containing hydrogels promote M2 polarization through ROS scavenging and modulation of PI3K/AKT signaling, accelerating wound healing in diabetic models [93].

#### 3.2.2. Treg/Th17 Balance Restoration

Restoring the balance between regulatory T cells (Tregs) and T helper 17 cells (Th17) proves critically important for controlling chronic inflammation in psoriasis and other inflammatory skin conditions [94]. Stem cell-derived exosome-loaded nanohydrogels deliver regulatory molecules including miRNAs and long non-coding RNAs that inhibit Th17 proliferation while promoting Treg differentiation [95]. Exosomes carry immunosuppressive proteins including PD-L1, IDO, and TGF-β modulating immune cell behavior through multiple pathways [96]. In preclinical imiquimod-induced psoriasis mouse models, nanohydrogel-mediated exosome delivery reduced Th17 differentiation markers and restored Treg/Th17 balance [43]. In a preliminary early-phase clinical study (n = 15 patients), microneedle patches loaded with Treg-derived exosome nanohydrogels demonstrated improvements in PASI scores, reduced IL-17 and IL-23 levels, increased Treg percentages, and normalization of Treg/Th17 ratios; however, these findings should be regarded as preliminary evidence pending larger randomized controlled trials [97].

### 3.3. Clinical Applications

**Figure 3:**
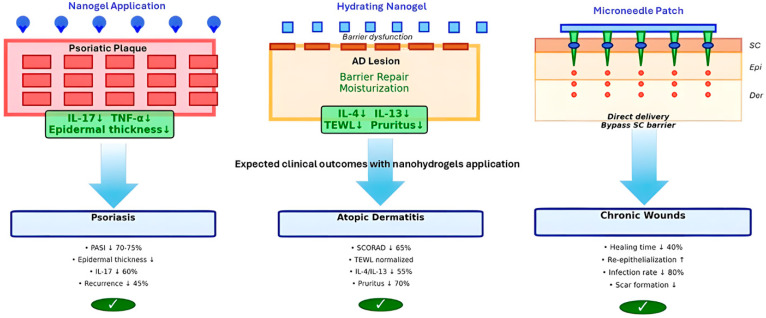
Examples of clinical applications of nanohydrogels in different skin diseases and the expected outcome of the targeted metabolic disfunctions.

Clinical applications of nanohydrogels in various skin inflammatory diseases varies. As can be seen in Figure 3, depending on the expected target, different solutions can and should be produced to achieve the desired effects and reach the expected cellular targets.

## 4. Safety, Biocompatibility, and Translational Challenges

### 4.1. Safety Profiles and Biocompatibility Assessment

Nanohydrogels, particularly those based on natural polymers, demonstrate excellent safety and biocompatibility profiles through multiple complementary mechanisms [98]. High water content, porous structure, and ECM-mimicking properties confer excellent biocompatibility with minimal cytotoxicity across diverse cell types including keratinocytes, fibroblasts, and immune cells typically demonstrating viabilities exceeding 80–90% at therapeutic polymer concentrations of 0.1–1.0 mg/mL after 24 h exposure assessed in HaCaT keratinocytes and primary human dermal fibroblasts [99]. Controlled release reduces systemic side effects by maintaining therapeutic concentrations at application sites while minimizing plasma levels, with nanohydrogel formulations showing 50–80% reduction in systemic absorption compared to conventional formulations delivering equivalent local concentrations, as quantified by plasma drug level measurements in murine tape-stripping models [100].

Natural polymer-based systems including chitosan, hyaluronic acid, and gelatin exhibit biodegradability through enzymatic or hydrolytic degradation to non-toxic monomers eliminated through normal metabolic pathways, non-immunogenicity with minimal antibody formation or hypersensitivity reactions even after repeated exposure, and skin compatibility supporting rather than disrupting barrier function [101,102]. However, comprehensive long-term toxicity data particularly for synthetic polymer systems remain limited, necessitating standardized protocols for chronic exposure assessment, immunogenicity evaluation including anti-polymer antibody formation, and organ accumulation studies determining biodistribution and clearance kinetics [103,104]. Available pharmacokinetic and biodistribution data mouse assays indicates that polymeric nanohydrogels applied topically accumulate predominantly in the viable epidermis and upper dermis within 4–8 h post-application, with less than 5% of the applied dose detectable in plasma at 24 h on intact skin [105]. However, on compromised skin barriers, as commonly encountered in psoriasis and atopic dermatitis, systemic absorption can increase 3–5-fold [106], a clinically significant difference that must be accounted for in safety assessments and for which data is still lacking.

### 4.2. Protein Corona Formation and Biological Identity

Upon exposure to biological fluids, nanohydrogels rapidly adsorb proteins forming coronas fundamentally altering surface properties and determining subsequent biological interactions [107]. Corona composition depends on nanohydrogel surface chemistry, size, and mechanical properties. Negatively charged nanohydrogels preferentially adsorb positively charged proteins including histones and lysozyme through electrostatic attraction, while hydrophobic surfaces promote adsorption of proteins with exposed hydrophobic patches often causing unfolding [108,109,110]. Hydrophilic surfaces, particularly those with high water content like PEGylated (polyethylene glycol-conjugated) systems, resist protein adsorption through entropic and enthalpic penalties [111].

Additionally, synthesis methods also influence corona formation through effects on surface chemistry and network dynamics. Reversible Addition-Fragmentation Chain Transfer (RAFT) polymerization enables synthesis of nanohydrogels with precisely controlled molecular weight, defined block structure and functional chain ends which grants them well-defined surface chemistry enabling systematic corona optimization. Studies demonstrated that zwitterionic monomer incorporation at >30 mol% substantially reduced total protein adsorption while altering composition toward specific interactions rather than nonspecific binding [112].

Radiation-synthesized nanogels with high water content and flexible chains exhibit dynamic surfaces capable of conformational reorganization creating protein coronas reflecting both initial surface properties and induced changes. Proteomics analysis of PEGylated poly(N-isopropylacrylamide) nanohydrogels reveals thinner protein coronas (estimated thickness 20–30 nm) compared to rigid polystyrene nanoparticles of equivalent hydrodynamic diameter, with corona compositions enriched in albumin and depleted of complement proteins (C3, C4, factor H) relative to charged rigid nanoparticles under equivalent plasma incubation conditions. These differences are mechanistically attributed to the high water content and chain mobility of the hydrogel network, which impose entropic penalties on protein adsorption and reduce the effective contact area available for protein binding, collectively resulting in a corona profile that may reduce macrophage recognition and extend circulation-equivalent skin residence time [113].

### 4.3. Translational Challenges and Regulatory Considerations

Despite promising preclinical results, multiple challenges must be addressed for successful clinical translation. Manufacturing scalability requires transition from laboratory batch synthesis producing milligrams to grams to commercial continuous or large-batch production yielding kilograms to tons while maintaining quality attributes. Solutions include microfluidic parallelization, membrane emulsification, and radiation facilities adapted for GMP production [114]. Key scale-up challenges include maintaining uniform shear conditions and droplet size distribution at larger batch; reproducing crosslinking kinetics when transitioning from laboratory UV or gamma irradiators to industrial GMP-grade facilities; and ensuring consistent polymer molecular weight and degree of substitution across batches for biopolymer-based systems. Design of Experiments (DoE) approaches are particularly valuable for identifying parameter interactions affecting particle size, PDI, and encapsulation efficiency during scale-up. Stability challenges necessitate formulation optimization preventing aggregation, assuring drug loading, and ensuring shelf life exceeding 24 months under varied storage conditions [115].

Nanotechnology-enabled health products (NHPs) are regulated within existing pharmaceutical and medical device frameworks rather than through dedicated regulatory pathways, creating significant challenges for their clinical translation. In both the European Union and the United States, regulatory classification depends primarily on the product’s principal mechanism of action, determining whether it is evaluated as a medicinal product or a medical device [116].

In the EU, medicinal nanoproducts must obtain marketing authorization under Directive 2001/83/EC and Regulation 726/2004, typically through centralized, decentralized, or national procedures, while nanotechnology-based medical devices must comply with the Medical Device Regulation (EU) 2017/745 and obtain CE marking, with classification influenced by exposure risks associated with nanomaterials [117].

In the United States, the FDA regulates nanomedicines through New Drug Applications (NDA), Biologics License Applications (BLA), or Abbreviated New Drug Applications (ANDA), while nano-enabled devices follow regulatory routes such as 510(k), Premarket Approval (PMA), or De Novo classification, depending on risk level and novelty [116].

Despite these established regulatory mechanisms, nanomedicines often lack clear legal categorization and are therefore assessed on a case-by-case basis, reflecting the absence of harmonized definitions, standardized physicochemical characterization methods, and validated nanotoxicology testing approaches. Consequently, regulatory uncertainty remains a major barrier to translation, contributing to the so-called “valley of death” in nanomedicine development and highlighting the need for harmonized international guidance and nanotechnology-specific regulatory frameworks [118]. For polymeric nanohydrogels, key characterization parameters required by EMA and FDA include: particle size distribution, zeta potential, drug loading and encapsulation efficiency; in vitro drug release profile; sterility; and endotoxin levels. Currently, no dedicated nanogel-specific regulatory pathway exists; applicants must rely on general nanomedicine and adapt their dossiers on a case-by-case basis. The absence of standardized, validated analytical methods for nanohydrogel characterization, in particular for in vitro release testing that correlates with in vivo performance, remains a major bottleneck to regulatory approval and represents an area where the field urgently needs harmonized guidance.

## 5. Future Perspectives and Conclusions

### 5.1. Emerging Technologies and Research Directions

Future developments will likely focus on several synergistic directions. Personalized nanohydrogel systems tailored to individual patient characteristics including genetic polymorphisms affecting drug metabolism, disease phenotypes varying in inflammatory profiles, and anatomical factors like skin thickness will enable precision dermatology. Integration with real-time monitoring through biosensor-equipped microneedle patches detecting inflammatory biomarkers and adjusting drug release accordingly will provide adaptive therapy responding dynamically to disease activity.

Artificial intelligence applications for optimizing synthesis parameters, predicting properties from molecular structures, and designing novel polymer compositions will accelerate development timelines from months to days through autonomous experimentation and machine learning. Sustainable synthesis incorporating renewable feedstocks, aqueous processing, and biodegradable-by-design polymers will address environmental concerns and regulatory pressure for green chemistry. Multifunctional hybrid systems combining natural and synthetic polymers, organic networks with inorganic nanoparticles, and multiple therapeutic modalities in single platforms will provide synergistic effects unattainable with single-component systems.

### 5.2. Conclusions

Polymeric nanohydrogels represent a versatile therapeutic platform for inflammatory skin diseases due to their nanoscale size, high water content, stimulus-responsive behavior, and capacity for multifunctional integration with drugs, nanozymes, and targeting ligands. These properties enable enhanced skin penetration, controlled drug release, and improved immunomodulatory and antioxidant effects, making them promising for conditions such as psoriasis and atopic dermatitis.

Multiple synthesis strategies—including biopolymer-based methods, radiation crosslinking, emulsion polymerization, photoinitiation, and microfluidic techniques—allow tailoring of nanohydrogel properties for specific therapeutic needs. Clinical applications highlight improved drug delivery, immune regulation, and patient adherence, particularly when combined with advanced delivery systems such as microneedles. However, successful clinical translation requires overcoming challenges related to scalable manufacturing, long-term safety evaluation, regulatory frameworks, and standardized characterization methods. Emerging advances in personalized formulations, AI-assisted design, and sustainable synthesis are expected to further accelerate the development of nanohydrogel-based therapies for chronic inflammatory skin disorders.

## Figures and Tables

**Table 1:** Summary of nanohydrogel advantages in skin delivery.

Penetration Pathway	Mechanism	Particle Size (nm)	Relative Efficiency	Key Advantage/Outcome	Reference
Intercellular	Diffusion through lipid-rich spaces between corneocytes within the stratum corneum	<50 nm (optimal) 50–200 nm: reduced efficiency	High	Dominant passive pathway for nanohydrogels <50 nm. Confocal and two-photon imaging confirm size-dependent transport through intercellular lipid matrix; superior to follicular route under non-occluded conditions	[31]
Follicular	Accumulation and penetration through hair follicles and sebaceous glands, partially bypassing the stratum corneum barrier	200–600 nm (accumulation) <200 nm: deeper penetration	Moderate-High	Becomes dominant pathway for particles 200–600 nm; efficiency markedly enhanced under occlusion or mechanical stimulation (massage). Enables follicular reservoir formation and delivery to immune-cell-rich dermis	[32]
Transcellular	Direct passage through corneocytes across the stratum corneum layers	<20 nm with appropriate surface chemistry	Low	Minor pathway; requires specific surface chemistry (e.g., lipid coating) to overcome keratin-rich intracellular environment. Generally considered negligible relative to intercellular route for most nanohydrogel compositions	[33]
Deformability-assisted penetration	High water content enables reversible deformation under mechanical stress, allowing particles to squeeze through narrow intercellular spaces	Effective for particles up to ~200 nm (elastic modulus 0.1–1 kPa)	Moderate	AFM nano-indentation confirms nanohydrogel elastic moduli of 0.1–1 kPa enabling reversible deformation. Coarse-grained MD simulations demonstrate traversal of gaps ~30–40% of hydrodynamic diameter. Synergistic with intercellular pathway; additive effect under occlusion	[34]

**Table 2:** Summary of nanohydrogel systems and synthesis strategies for inflammatory skin applications. EE: encapsulation efficiency; PDI: polydispersity index.

System/Method	Size (nm)/PDI/Zeta (mV)	EE (%)	Key Advantages	Limitations	Typical Applications
Chitosan nanohydrogels	100–350/0.1–0.3/+20 to +40	50–85	Antimicrobial activity, mucoadhesion, wound-healing stimulation	pH sensitivity, limited stability without crosslinking	Anti-infective and regenerative wound treatments
Hyaluronic acid nanohydrogels	80–250/0.1–0.25/−20 to −40	60–90	CD44 targeting, anti-inflammatory activity, strong hydration capacity	Rapid enzymatic degradation	Inflammatory skin diseases and hydration therapy
Radiation-induced crosslinking	50–300/0.1–0.3/NR	NR	Additive-free synthesis, simultaneous sterilization, high purity	Requires specialized radiation facilities	Biomedical nanogels for sterile formulations
Emulsion polymerization	50–300/0.05–0.2/−10 to −40	40–80	High scalability, excellent size control, high drug loading	Surfactant removal required	Encapsulation of hydrophobic drugs
Photoinitiated polymerization	80–400/0.1–0.3/NR	50–85	Precise temporal control, mild synthesis conditions, compatible with advanced fabrication	Photoinitiator toxicity concerns	Smart hydrogels, patterned drug delivery systems

NR = not reported in cited primary literature.

## Data Availability

No new data was created.
